# Chlamydia and HIV testing, contraception advice, and free condoms offered in general practice: a qualitative interview study of young adults’ perceptions of this initiative

**DOI:** 10.3399/bjgp17X691325

**Published:** 2017-05-23

**Authors:** Leah Ffion Jones, Ellie Ricketts, Katy Town, Claire Rugman, Donna Lecky, Kate Folkard, Anthony Nardone, Thomas Nathan Hartney, Cliodna McNulty

**Affiliations:** Sexual Health Promotion, Public Health England, Primary Care Unit, Gloucester Royal Hospital, Gloucester.; Sexual Health Promotion, Public Health England, Primary Care Unit, Gloucester Royal Hospital, Gloucester.; Sexual Health Promotion, Public Health England, Primary Care Unit, Gloucester Royal Hospital, Gloucester.; Sexual Health Promotion, Public Health England, Primary Care Unit, Gloucester Royal Hospital, Gloucester.; Sexual Health Promotion, Public Health England, Primary Care Unit, Gloucester Royal Hospital, Gloucester.; Sexual Health Promotion, Public Health England, Primary Care Unit, Gloucester Royal Hospital, Gloucester.; Sexual Health Promotion, Public Health England, Primary Care Unit, Gloucester Royal Hospital, Gloucester.; HIV/STI Department, National Infections Service, Public Health England, Primary Care Unit, Gloucester Royal Hospital, Gloucester.; Primary Care Unit, Gloucester Royal Hospital, Gloucester, and visiting honorary professor, Cardiff University, Cardiff.

**Keywords:** chlamydia screening, general practice, HIV testing, patient preference, primary health care, sexual health, young people

## Abstract

**Background:**

Opportunistic chlamydia screening is actively encouraged in English general practices. Based on recent policy changes, Public Health England piloted 3Cs and HIV in 2013–2014, integrating the offer of chlamydia testing with providing condoms, contraceptive information, and HIV testing (referred to as 3Cs and HIV) according to national guidelines.

**Aim:**

To determine young adults’ opinions of receiving a broader sexual health offer of 3Cs and HIV at their GP practice.

**Design and setting:**

Qualitative interviews were conducted in a general practice setting in England between March and June 2013.

**Method:**

Thirty interviews were conducted with nine male and 21 female patients aged 16–24 years, immediately before or after a routine practice attendance. Data were transcribed verbatim and analysed using a thematic framework.

**Results:**

Participants indicated that the method of testing, timing, and the way the staff member approached the topic were important aspects to patients being offered 3Cs and HIV. Participants displayed a clear preference for 3Cs and HIV to be offered at the GP practice over other sexual health service providers. Participants highlighted convenience of the practice, assurance of confidentiality, and that the sexual health discussion was appropriate and routine. Barriers identified for patients were embarrassment, unease, lack of time, religion, and patients believing that certain patients could take offence. Suggested facilitators include raising awareness, reassuring confidentiality, and ensuring the offer is made in a professional and non-judgemental way at the end of the consultation.

**Conclusion:**

General practice staff should facilitate patients’ preferences by ensuring that 3Cs and HIV testing services are made available at their surgery and offered to appropriate patients in a non-judgemental way.

## INTRODUCTION

Chlamydia is the most common sexually transmitted infection in Europe and rates among young people are increasing.^1^ Testing those at higher risk of chlamydia infection and treating them effectively could reduce sequelae and onward transmission. General practice provides an ideal venue for opportunistic chlamydia testing when young people visit their GP practice.^2^ GPs have performed a smaller proportion of all chlamydia tests undertaken compared with other sexual health providers (genitourinary medicine and sexual and reproductive health care [GUM and SRH])^3^ despite research showing that primary care can provide an effective, popular alternative to GUM and SRH clinics.^4^^,^^5^ National guidance in England produced by the National Institute for Health and Care Excellence (NICE) and the British HIV Association *et al* 6 considers that in areas of high HIV prevalence (two or more in 1000 population), an HIV test should be offered when a patient registers at a practice or a patient has an indicator illness.^6^ Indeed, opt-out HIV testing is regarded as appropriate and acceptable when offered to patients registering at a practice.^7^

The theory of planned behaviour^8^ is used to explain human behaviour and has been used to design interventions to change primary care staff intentions when implementing evidence-based practice. A complex intervention carried out by Public Health England (PHE) in 2010 (the Chlamydia Intervention Randomised Trial [CIRT]) aimed at increasing opportunistic chlamydia testing in GP practices. It used the theory of planned behaviour to influence attitudes, subjective norms, and perceived behavioural control, and proved successful (success was measured by a sustained change in chlamydia screening and diagnosis).^9^ Therefore, an updated intervention was planned by PHE to include a broader opportunistic sexual health offer including chlamydia testing, contraception advice, sign-posting to free condoms, and HIV testing where appropriate — this is referred to as 3Cs and HIV. The authors aimed to expand on the previous research and use qualitative methods to explore patients’ attitudes to this wider 3Cs and HIV offer, using the theory of planned behaviour to provide an understanding of any potential facilitators or barriers to implementing this intervention.

## METHOD

### General practice selection

The authors chose areas in England (Bournemouth and Poole, Warwickshire, and Plymouth) to obtain a mix of deprivation, ethnicity, and rural and city locations. GP practices in each area were ranked into three categories by their 2012 chlamydia screening rate. Six practices in the highest tercile were randomly selected and approached in order. Six surgeries were purposively selected from the lowest tercile that were closest geographically to the high testing surgeries. Practices were approached by letter and telephone for consent to approach their patients (35 practices were approached by telephone — seven refused, 12 agreed, and 16 were not followed up as saturation was reached]. Piloting was conducted in Islington as it was geographically convenient for the researchers to meet.

**How this fits in**

Young people find general practice an acceptable venue for chlamydia testing; they report that being offered an opportunistic chlamydia screen is easier than having to ask for one, and that it gives them the opportunity to ask questions. This study extends beyond previous research by examining young adults’ perceptions of being offered contraception, condoms, and, where appropriate, an HIV test (3Cs and HIV) in addition to the offer of a chlamydia test when visiting their GP practice. This research will help inform future decisions around developing sexual health services in primary care.

### Participant recruitment

All interviews were completed over 11 days. Practices provided researchers with an anonymised list of patients within the 16–24 age bracket attending the surgery that day and their appointment time, in order to assist researchers in identifying potential participants. Researchers approached patients in the waiting area of 11 general practices (irrespective of their reason for attendance) from March to June 2013 and invited them to participate in a face-to-face interview in private. (The results could not be published sooner because this study relates to another study using a McNulty–Zelen design.)^10^ When researchers approached potential participants, they introduced themselves and explained the nature of the study.

The first question they asked was to see if the patient was aged 16–24 years, and, if so, they offered the patient an interview and provided them with information. If a potential participant was not aged 16–24 years the researcher explained that they did not meet the criteria for the study and provided further information if requested. Face-to-face interviews were chosen in order to maximise convenience for the participants and therefore facilitate recruitment.

All participants were assured of anonymity and confidentiality, and gave written informed consent. Patients received a £10 voucher for their time.

### Interviews

**Figure 1.**
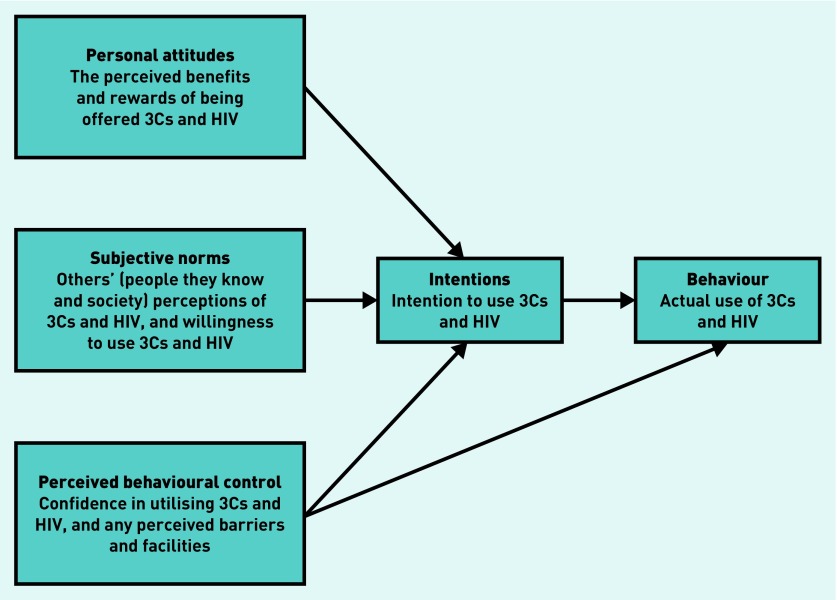
***Theory of planned behaviour: perceptions of an offer of chlamydia testing with providing condoms, contraceptive information, and HIV testing.***

The interview schedule (Appendix 1) was based on previous research examining attitudes towards, and preferences for, chlamydia screening,^5^ and was agreed by a steering group. The steering group was an advisory group for the National Chlamydia Screening Programme in the delivery of the 3Cs and HIV project. The purpose of the group was to ensure the 3Cs and HIV project is developed and delivered in a way that is relevant, supportive of, and appropriate for primary care, and to ensure primary care ownership of the 3Cs and HIV project. Its purpose was also to ensure that decisions made by the project group were informed by the reality of primary care practice on the ground and to inform the content and style of 3Cs and HIV intervention. Members of the steering group include a service user, GPs, and practice nurses, and was piloted with three patients. The final semi-structured interview schedule followed the broad topic areas within the theory of planned behaviour’s conceptual framework in order to understand the influences on behaviour (Figure 1).^11^ The broad areas discussed included:

- interviewees’ attitudes towards being offered opportunistic chlamydia screening, contraception, condoms, and HIV tests at a GP practice (attitudes);
- perceived staff and friends’ attitudes (subjective norms);
- perceived barriers and self-efficacy (perceived behavioural control); and
- opinions on receiving 3Cs and HIV.

Three researchers conducted the interviews. Participants were aware that the researchers were not affiliated with the GP practice and were encouraged to discuss their opinions freely. Interviews lasted 20–30 minutes, were audio-recorded, transcribed verbatim, and checked for accuracy. Data were analysed by a fourth researcher using a thematic framework in NVivo (version 10).

Data saturation was reached, themes were refined, and redundant or infrequent codes were recoded. One-third of transcripts were double coded by a second researcher. Codes were discussed and an agreed consensus was reached on an appropriate framework.

## RESULTS

Thirty interviews were conducted with nine male and 21 female patients, aged 16–24 years. Eighteen participants were aged ≥21 years and 12 participants were aged 16–20 years. None of the participants objected to any questions and all interviews were completed in full.

There were no obvious differences in opinions or attitudes between locations or age of participants. The only differences observed were between female and male preferences for method of chlamydia testing and the sex of staff, as described below.

### Patient preferences for location, method, and member of staff

Several participants indicated that they had never been offered a chlamydia test or discussed contraception with their GP. However, nearly all participants interviewed would be happy to be screened for chlamydia in their GP practice:

‘If I was concerned about chlamydia, I’d rather do it at my GP’s surgery because my GP knows me and I’d feel more sort of comfortable discussing options with them, and knowing that they know my history and stuff like that.’

(Patient 2, female [F], age 24 years, Islington)

Many would be happy and indeed prefer to discuss contraception with their GP, although several said they preferred to purchase condoms from a shop:

‘You’d rather have your doctor give it to you, or say you know, here this is a private room no one’s around, you know, it’s easier to get condoms, and you’d feel more comfortable getting condoms or talking about contraception here than to go a health clinic, wait up, queue up. Umm, and just ask them, “Can I have some condoms?”’

(Patient 18, male [M], age 16 years, Warwickshire)

Furthermore, most participants reported being happy to receive an HIV test as part of a new patient registry, as a result of an indicator illness, or as a result of a sexual health background discussion.

Several participants said they would not mind being offered a chlamydia screen in an unrelated consultation, and a few identified the importance of this as it captures those that would not raise the topic themselves:

‘Probably preferable here, it’s more convenient, you can sort of kill two birds with one stone, see the doctor and get two things done at once.’

(Patient 8, F, age 23 years, Bournemouth and Poole)

Several participants reported that being offered an HIV test was a good precaution to take because they would not want to go undiagnosed:

‘I’d think that’s fine, like it’s doctor patient confidentiality, you know, it is about your health and it’s better to just to open up so that they can help you.’

(Patient 15, F, age 24 years, Islington)

One male participant preferred attending a sexual health clinic over his GP practice for chlamydia screening and contraception advice because its location was convenient:

‘And you can go in your lunch break, and it was just convenience basically … Yes, it’s not really that I wouldn’t go to my GP, I would but … it’s just often with GPs you know it takes longer ... to get an appointment, and that’s basically it.’

(Patient 14, F, age 21 years, Islington)

Preferences for the method of chlamydia test, staff member, and the sex of staff all elicited mixed views. Although several female participants preferred the self-taken swab method, a few preferred providing urine. Several preferred taking the chlamydia test straight away rather than taking it home because they reported that they might forget to return it, did not want to carry the sample around with them, and did not like the idea of posting the sample back. Some did not mind either way:

‘I would, I don’t really think I’d have a preference for it, I wouldn’t mind.’

(Patient 15, F, age 24 years, Islington)

Some were happy for the receptionist to offer the chlamydia test, whereas some indicated that it might not be appropriate because of the practice layout:

‘I think the receptionists … once again I think it depends on the area of the surgery, or like the layout maybe, because I don’t think the new medical centre … you’ve got from, this is the couch and there’s about a metre, two metres before the reception desk, and I think it all depends on the layout of the room.’

(Patient 1, M, age 24 years, Islington)

Preference for the staff member appeared to be related to the patient’s sex because some females would prefer a female clinician, although few mentioned this at all:

‘Being a guy, I think you’d want to see a guy for it, and probably most ladies would want to see a female doctor for it.’

(Patient 7, M, age 22 years, Bournemouth and Poole)

‘I think if I was doing an actual chlamydia test, I’d rather it was a female, but in terms of contraception I wouldn’t really mind.’

(Patient 13, F, age 24 years, Plymouth)

‘I think probably nurse I’d feel most comfortable with, because it’s usually the nurse who I have an appointment with to get the contraceptive pill, so I feel I’d probably feel sort of most comfortable with that.’

(Patient 6, F, age 23 years, Islington)

### Other important factors for patients

#### The convenience of taking a test

The most important factor to participants was convenience, described by most as being facilitated by proximity to the test location, having a range of chlamydia testing methods available, having a simple procedure, and patients having enough knowledge about the testing procedure to facilitate the whole process:

‘At our medical centre there’s a box, a big like box, with chlamydia tests in the bathroom, which I think’s a good idea because then when you’re in there you’re kind of, oh I can do this while I’m here.’

(Patient 1, M, age 24 years, Islington)

‘It’s good anyway, you know for it to be available in as many places as possible.’

(Patient 14, F, age 21 years, Islington)

‘They usually always ask me to take the chlamydia swab into the toilet, and they explain the instructions to me before I go in, and, uh, once you’re in the toilet there’s instructions posted on the back of the wall, and just to remind you what you need to do and yeah, and that’s helpful as well because I get quite nervous about whether I’m doing it properly.’

(Patient 17, F, age 23 years, Bournemouth and Poole)

#### The offer of testing should be routine

Many participants reported that sexual health should be raised routinely in consultations. Thus, participants reported that it was important for staff to indicate that the offer was made because the patient met the target demographic, as it eliminates the perception of being given the offer due to a suspected likelihood of having a sexually transmitted infection:

‘It should be, it should be more openly spoken about, I think.’

(Patient 5, F, age 18 years, Islington, on discussing chlamydia and contraception with a GP)

Interviewer: ‘*How should the staff ask you to do the* (chlamydia) *test?’*

Patient: ‘Ummm, I suppose not in a way that it sounds like you’ve been sleeping around, like.’

(Patient 21, F, age 21 years, Islington)

*‘Yeah, I mean, if they just offered it to everyone at that age then you know that you haven’t been singled out, you know, it’s not an embarrassing thing to … you can’t change your age sort of thing* [laughs]*, it’s not because of who you are or what they think, and sort of thing.’*

(Patient 12, F, age 20 years, Bournemouth and Poole, on 3Cs and HIV)

‘I think they need to phrase it as part of a like a drive screening drive, that it wasn’t a personal thing.’

(Patient 29, M, age 21 years, Islington, on chlamydia screening)

#### Reassurance around testing is key

Participants felt that an important part of being offered an HIV test is having the *‘right kind of doctor’* to discuss HIV with, in addition to having support available for positive results. Several participants felt that the topic of HIV should be broached carefully to avoid unnecessarily alarming patients. This issue was stressed particularly when participants were provided with examples of HIV indicator illnesses and conditions:

‘You need someone to kind of profile that person’s risk factor before they do that test, because if there’s a chance of it being positive you kind of need to offer support with it as well.’

(Patient 29, M, age 21 years, Islington)

‘Fine, absolutely fine, it might be a bit worrying at first but as long as you’re reassured that you know, that necessarily wasn’t the case, it’s just routine I’d be fine.’

(Patient 19, M, age 16 years, Warwickshire)

### Barriers and perceived barriers to discussing and accepting an offer of 3Cs and HIV

#### Embarrassment and unease around testing

Participants said they themselves or others do not necessarily want to discuss chlamydia screening or contraception in a non-sexual health consultation, or would worry that their parents may find out. Other situations identified as causing embarrassment and unease included having to walk through reception with a urine sample or taking the test in front of a GP, or if the GP sees their family members:

‘I’d probably do it … it’s not, you’re just screening but I think sometimes people are embarrassed by the fact that they then have to walk through the waiting room with a pot.’

(Patient 1, M, age 24 years, Islington)

‘Umm, good actually, because umm I’ve been to get one and it’s quite embarrassing to ask someone, so if they offer it it’s easier just to say “yeah”.’

(Patient 8, F, age 23 years, Bournemouth and Poole, on chlamydia screening in GP practices)

‘I think free condoms should be offered routinely for anyone that’s in the sort of teenage to twenties age bracket, ummm because people might be embarrassed to ask about them and might be less likely to go to the GP about that kind of thing.’

(Patient 2, F, age 24 years, Islington)

Patient: ‘Should be OK unless, I don’t know, if my parents were around it would be a bit awkward.’

Interviewer: ‘Ok yep and do you go to the surgery often with your parents?’

P: ‘Most of the time it is with my parents, yeah.’

(Patient 4, M, age 19 years, Islington, on chlamydia screening in GP practices)

‘For a urine test, I mean when I’ve had to do them here before for different things, ummm, it is a bit, you’ve got to walk past the really busy reception with your … with your wee sample or something, but I mean that’s a problem in a lot of places.’

(Patient 12, F, age 20 years, Bournemouth and Poole)

‘Again, it would feel a little bit … quite strange for my GP suddenly, just talked about where I could get condoms from at the end of, umm, a consultation, ummm, but it would just feel sort of … a bit random.’

(Patient 29, M, age 21 years, Islington)

‘Yeah, I think it has to be done really, it may be a bit embarrassing at first for some people but I think it has to be done.’

(Patient 6, F, age 23 years, Islington, on HIV testing)

Some participants perceived that for other patients it may be inappropriate for GP staff to raise 3Cs and HIV testing in an unrelated consultation if the patient was religious, as it could cause offence:

‘Some might … not use I don’t know, maybe just religious reasons.’

(Patient 3, F, age 24 years, Islington)

‘Yeah, I think some people might be, may be offended if they were asked you know, people who never had sex or whatever, or like people who are religious.’

(Patient 9, M, age 20, Bournemouth and Poole)

‘I think I’d be a bit offended if my GP just said I think you need to consider whether you’ve got chlamydia or not.’

(Patient 29, M, age 21 years, Islington)

‘Oh I don’t know, I think they should check it’s not that first, I don’t know. I don’t know, because I know some people would take major offence to that, I know a lot of people if you said “Oh you might have HIV” they’d kick off and be like “You know, what you trying to say?”’

(Patient 7, M, age 22 years, Bournemouth and Poole)

#### Time

A few participants were concerned that having a chlamydia screening or contraception discussions in an unrelated consultation would take time away from their original reason for visiting the GP, reducing the quality of that consultation, or result in the need to book an additional appointment. Some participants felt that 3Cs and HIV should be offered if there is time at the end of the consultation to avoid this:

‘I just think make it at the end of the appointment to say, ummm yeah, “We’re doing this national screening programme because chlamydia’s really quite common in your age group, do you just wanna do the screening?”’

(Patient 29, M, age 21 years, Islington)

‘You know, killing two birds one stone … when you see the doctor once every six months say get checked.’

(Patient 8, F, age 23 years, Bournemouth and Poole, on chlamydia testing)

‘So I think it should just be added on, obviously deal with the problem first whatever it may be, cold, flu, whatever it is, and just add it on.’

(Patient 5, F, age 18 years, Islington, on chlamydia screening and contraception)

‘Well because, this I think, this clinic is quite busy, so usually an appointment’s sort of 3/4 weeks you know … I think I saw them a month ago so it’s a bit bad timing for exams, but like I could see them afterwards I suppose.’

(Patient 15, F, age 24 years, Islington)

‘Doing a urine sample there and then is, ummm, is just extra GP time and maybe that would take away from time you need to actually talk about the problem that you came in for.’

(Patient 29, M, age 21 years, Islington)

Two participants said it would be useful to have the issue of sexual health raised at every visit:

‘I suppose if it is raised each time then, and they are sure you’re up to date, and that sort of thing, it would become a part of the consultation I suppose, it’d be a normal thing to be asked until you’re older, sort of thing, yeah.’

(Patient 12, F, age 20 years, Bournemouth and Poole, on chlamydia screening)

### Facilitators and suggestions for raising awareness and highlighting the importance of trust and confidentiality

#### Raising awareness of sexual health services

Two individuals felt that awareness should be raised around GP practices offering sexual health services:

‘Just say “We offer chlamydia screening here”, or I don’t know, “When was the last time you had a check up, like sexual health check up?”’

(Patient 9, M, age 20 years, Bournemouth and Poole)

Indeed, some participants were not aware that they could get a chlamydia test at their GP practice:

‘There could be a bit more awareness I suppose of where people can go.’

(Patient 23, F, age 17 years, Islington)

One individual stated that they would be pleased to know that they had been offered all the sexual health services available from their GP.

One female highlighted the importance of discussing all contraception options along with condoms:

‘So I think it’s better to outline the different options and let the patient make up their mind about which one’s best.’

(Patient 6, F, age 23 years, Islington)

Several reported that posters in waiting rooms can raise awareness; however, a few reported that they do not look at posters as there are often too many:

*‘I think even like in a lot of surgeries, just waiting around I like to read everything on the wall and stuff, but there’s never really much about things like that, there’s never really anything about … unless you go to a GUM clinic then it’s* e*verywhere.’*

(Patient 7, M, age 22 years, Bournemouth and Poole)

#### Trust in GP staff, and reassuring confidentiality

Several participants mentioned the benefits of having trust in their GP or their GP practice, and a few placed a high value on the professionalism of their GP when discussing sexual health:

‘I think it’s probably a combination of the fact that people are embarrassed to talk about sexual health, GPs don’t necessarily bring it up every time so they may not know it’s available, and, ummm, I think that kids might be worried that it’s gonna go on their record, that they asked for this or that, they’ve talked about it and that their parents will find out. That they’re sort of sexual active when they … want to conceal that, umm, so I think it’s probably a combination of factors, I think GPs should make it clear to everyone when they offer them the chlamydia test that it’s confidential and they’re not going to go and tell their parents about it.’

(Patient 2, F, age 24 years, Islington)

‘So, urm, I think, umm, discretion and support and explaining it properly with the person.’

(Patient 23, F, age 17 years, Islington, on chlamydia screening)

‘I’ve been coming to this surgery for a long time and I trust all my doctors and everyone that I’ve met in this surgery, feels like a family which I can trust.’

(Patient 28, F, age 24 years, Warwickshire)

‘I think it might just be my experience with them has been really opening and helpful, that I think I wouldn’t feel judged by them.’

(Patient 1, M, age 24 years, Islington)

‘I would definitely like to have the screening at the GP because I trust them more than anywhere else.’

(Patient 28, F, age 24 years, Warwickshire)

### Knowledge of chlamydia, screening, chlamydia treatment, contraception, and sexual health services

Nearly all participants knew at least one fact about chlamydia and screening: around where to obtain a test, duration to receive results, or methods of testing:

‘I know, it’s well, you screen people for chlamydia like, test to see if they have chlamydia, and I think you can use, umm, swabs, and that’s all I really know about it.’

(Patient 15, F, age 24 years, Islington)

‘Umm, I know it’s done by a nurse, and I know that you can come in, get it done and get the results in a few days.’

(Patient 27, F, age 22 years, Warwickshire, on chlamydia tests)

‘Chlamydia screening is when you, umm, just run a test, with kind of as many people as possible, just to try and pick up any cases that might be undetected.’

(Patient 29, M, age 21 years, Islington)

Interviewer: ‘Is that a test you’ve had before?’

Patient: ‘Yeah, I’ve had that before’

I: ‘How long did you wait for your result?’

P: ‘Uh, about, no it was straight away, it’s like 5 minutes.’

(Patient 14, F, age 21 years, Islington, on HIV tests)

Others were less knowledgeable. Several participants had a lack of understanding on what chlamydia or a chlamydia screening was, treatment for chlamydia, and which health service to use if one was presenting with symptoms, while two individuals did not know how to obtain condoms:

‘I mean, even I don’t really know what chlamydia is and I’m 24, so a lot of young people don’t know.’

(Patient 2, F, age 24 years, Islington)

‘Ummm, what is a chlamydia screening?’

(Patient 3, F, age 24 years, Islington)

Interviewer: ‘Do you know what the screening means?’

Patient: ‘Not really, is it a test or something?’

(Patient 20, F, age 24 years, Islington)

Interviewer: ‘Do you understand the word chlamydia at all?’

Patient: ‘Not, not exactly, I’ve sort of, the word sounds familiar but yeah, I’m not entirely sure of the meaning.’

(Patient 25, M, age 17 years, Warwickshire)

‘I don’t know where they are to be honest, I don’t know where there is one.’

(Patient 8, F, age 23 years, Bournemouth and Poole, on sexual health clinics)

Interviewer: ‘Have you been to places like that?’

Patient: ‘No, what would they do?’

(Patient 5, F, age 18 years, Islington, on sexual health clinics)

‘Jeez, didn’t realise it was that high, I thought HIV was really rare.’

(Patient 7, M, age 22 years, Bournemouth and Poole, on prevalence of HIV)

‘I don’t know, I don’t really understand HIV to be honest.’

(Patient 10, F, age 23 years, Bournemouth and Poole)

## DISCUSSION

### Summary

Young people were open to being approached routinely in general practice about their sexual health. Perceived behavioural control (the perceived ease or difficulty of a particular behaviour) was the most influential aspect of the theory of planned behaviour. Convenience was a key factor, meaning that participants indicated a clear preference for receiving 3Cs and HIV, where appropriate, at their GP practice as long as it was done in a confidential and non-judgemental manner. Because of a reported lack of advertising, participants were not fully aware of all the sexual health services available in their GP practice and the exact testing process.

### Strengths and limitations

This is the first study exploring patients’ views on a combined offer of chlamydia screening, contraception, and HIV testing in GP practices.

Recruiting participants opportunistically at practices in areas varying in urban and rural location, ethnic diversity, deprivation, and rates of chlamydia testing allowed for the recruitment of a wide range of young adults with a range of attitudes and opinions. Despite not asking participants about their own sexual health, many offered this information without prompting, describing a range of behaviours from those that were accessing sexual health services regularly, to those with little or no experience in accessing any type of sexual health service.

Participants’ religion, ethnicity, and socioeconomic status were not collected in this study. Religion and ethnicity were identified as potential barriers to accessing sexual health services although no one identified this as a personal barrier. Research has demonstrated that a person’s religion can influence the behavioural and medical treatments for sexual complaints.^12^ Therefore, further research needs to be conducted to explore and understand this potentially influential barrier.

The face-to-face method of data collection may have restricted participants from expressing their views openly compared with telephone interviews; however, the data do not reflect this. Interviews were conducted in a private environment to reassure patients of confidentiality and anonymity, and to facilitate open discussion.

Practice staff gave researchers an anonymised list of patients attending on the day between the ages 16 and 24 years, and, out of the 32 potential participants provided on the lists, 30 were recruited (a 94% success rate). Therefore it was simply by chance that more females attended the practices on the days of recruitment. This is arguably a limitation of the study that the sample did not equally represent males and females. However, this is unsurprising because research has shown that females attend their GP practice more frequently than males and is therefore an accurate representation of the sex profile of people visiting their GPs.^13^

### Comparison with existing literature

Guidance from NICE^14^ and the British HIV Association *et al*^6^ considers that, in areas of high HIV prevalence, an HIV test should be offered when patients register at the practice. Research has shown that sexual health services in primary care can provide an effective, accessible, and popular alternative to GUM and SRH clinics.^4^^,^^5^ Participants indicated that they want sexual health services to be flexible and as accessible as possible while also providing a wide range of testing methods to suit all preferences. This is substantiated in a systematic review of chlamydia testing,^15^ showing that women want a range of options including urine tests, self-administered swabs, pelvic exams, clinician-collected swabs, home-testing, and community-based testing. The systematic review also indicated that patients considered that tests should be free, easy, and quick, thus demonstrating the importance of convenience and catering to patients’ preferences.^14^

The current study found that there was a clear lack of essential knowledge around chlamydia, and testing for it, as well as contraception, and HIV. Similar findings were reported in a questionnaire study examining chlamydia knowledge in men and women in Scotland,^16^ which found that, although awareness was high, knowledge decreased as questions became increasingly focused. Additionally, the National AIDS Trust reported that there are considerable knowledge gaps among the general public when it comes to the impact of HIV, HIV treatment, and transmission.^17^

A study asking men about an internet-based Sexually Transmitted Infections screening approach raised similar barriers to the sample in the current study about concerns of confidentiality.^18^ Similarly, young adults asked about factors and mechanisms that influence risky sexual behaviours identified concerns about confidentiality and stigma of accessing sexual health services.^19^

### Implications for practice

Extending chlamydia screening to include 3Cs and HIV in general practice appears to be acceptable to patients and could be implemented if appropriate resources are available, with readily accessible tests, patient information, and willing clinicians. Research is needed to fully understand the role of religion in the acceptability of receiving a 3Cs and HIV offer in general practice.

General practice staff need to align their 3Cs and HIV options with their patients’ preferences, and to raise awareness among young people of these options, including making patients aware that they can choose a female or male clinician, and explaining the testing methods available and where testing can be performed (at home, immediately in the practice, behind a curtain, in the toilet, or in another room).

As privacy was important to participants, it may not be appropriate for the receptionist to offer 3Cs and HIV to patients, unless confidentially can be ensured. Normalising the offer of 3Cs and HIV by emphasising that the offer is routine and given to all patients within the demographic would facilitate patients’ understanding of why they are being offered the test.

Patients lacked essential knowledge around sexual health. This could be improved by offering 3Cs and HIV and by providing more information during consultations. Despite participants displaying an overall understanding of the seriousness of HIV, a lack of overall knowledge means that reassurance by a professional is crucial for patients’ understanding of the implications of receiving a positive result.

This study suggests that educational interventions are required to increase young people’s understanding of 3Cs and HIV to inform their assessment of personal risk, which in turn may inform and change their health-seeking behaviours and sexual health risk behaviours.

## Appendix 1. Patient interview schedule

### Interview information (to be read by interviewer)

We are consulting patients aged 16–24 years at GP practices to determine whether having sexual health discussions during consultations on a routine basis are acceptable and if there are any barriers/issues that need to be addressed. We will be using the information gathered during these interviews to further develop the approach to chlamydia screening and sexual health in England within GP practices.

We wish to record the interviews to enable us to analyse full and accurate transcripts of what was said — the data will be anonymous and confidential. We will send you a transcript if you wish in order to comment on the interview at a later stage.

### Chlamydia screening

1. Can you tell me what you understand by the term chlamydia screening?
  - At-risk group may not know what screening is
2. What are your views on being offered a chlamydia screen in this surgery?
  - Do you think it is relevant to you?
  - If NO: Why not, what would make it more relevant? Probe more with men
3. Have you ever been offered a chlamydia screen?
  - If yes, when did you have your last screen?
4. If you were offered a chlamydia screen at the surgery today how would you respond?
5. Could you tell me about some other places where you might consider having a chlamydia screen?
  - Preference for surgery, family planning clinic, or other
6. Are there any advantages or disadvantages in coming to this surgery rather than going to Family Planning or Brook clinics for a chlamydia screen?
  - May refer to places mentioned in question 5
7. What do you think doctors and nurses at this surgery think about chlamydia screening?
8. For what reason do you think chlamydia screening should be offered in this surgery?
9. Some doctors and nurses ask patients to give a urine sample or self-taken swab for a chlamydia screen immediately in the surgery, rather than giving them the kit to take home. How would you feel about this? How do you think staff should ask for this sample to be done immediately?

### Contraception

1. Where have you discussed contraception with a doctor or nurse in the past year?
  - Probe whether raised by patient or practitioner
2. If not the surgery, why do you go for contraception advice elsewhere?
3. Are there any advantages or disadvantages in coming to the surgery rather than going to Family Planning or Brook clinics for contraception advice?
  - Link to previous answer
4. How would you find discussing chlamydia and contraception at the same time?
5. How would you find discussing chlamydia and contraception with different members of staff?
  - GP, nurse, receptionist
6. What would your family, partner, or friends think of you having a chlamydia screen and obtaining contraception advice (a basic sexual health check) in this surgery?
7. Are there things that would make it difficult for you to ask for contraception advice and have a chlamydia screen in this surgery?
8. Are there things that would make it easier for you to obtain contraception and have a chlamydia screen in this surgery?
9. How do you think staff should raise chlamydia and contraception with young people in this surgery?
11. What are your views on being offered contraception or a chlamydia screening test during a consultation that is not for contraception?
  - For example, what would you feel if you came in with a sore throat or any other illness and were asked if you would like contraception advice or a chlamydia screen?
12. What do you think about the idea of the doctor or nurse you see ***always*** offering a basic sexual health check when you attend?
13. Why might young people not always use contraception services or take up the offer of a chlamydia screen in this surgery?
14. How young-person friendly is this surgery?
  - *Ask about information displayed in the waiting room**, posters, leaflets*

### Condoms

1. What are your views about the surgery offering advice about where you may obtain free condoms?
  - How do you think this should be done? Who do you think they should ask? How do you think it could be raised in a consultation
2. What sort of consultation do you think this could be raised in?
3. What are your views about the surgery offering all young adults a small card (show card from study) about its services for contraception, chlamydia screening, and STIs?
4. How would you feel about a GP or nurse offering you condoms during your consultation?
8. For what reason do you think chlamydia screening should be offered in this surgery?
9. Some doctors and nurses ask patients to give a urine sample or self-taken swab for a chlamydia screen immediately in the surgery, rather than giving them the kit to take home. How would you feel about this? How do you think staff should ask for this sample to be done immediately?

### HIV

#### Interviewer mention national guidelines on HIV testing

1. How would you feel about being offered a routine HIV test at new patient registration appointment?
2. Guidance also suggests testing for HIV if a patient presents for specific indicator conditions. How would you feel about being offered a routine HIV test for an indicator disease (for example, glandular fever or shingles), even though these are common diseases with a low likelihood of being the result of an HIV infection?
3. What are your views on a doctor or nurse at this surgery asking you questions to decide whether you may be at risk of HIV?

#### Summarise discussion

Thank you very much for taking the time to participate in this interview.
